# Long-Term Clinical Study on Sandblasted–Acid-Etched Surface Dental Implants: 12-Year Follow-Up

**DOI:** 10.3390/ma18010183

**Published:** 2025-01-04

**Authors:** Eugenio Velasco-Ortega, Jesús Pato-Mourelo, Borja López-López, Loreto Monsalve-Guil, Jesús Moreno-Muñoz, José López-López, Enrique Núñez-Márquez, Nuno Matos Garrido, José Luis Rondón-Romero, Álvaro Jiménez-Guerra, Iván Ortiz-García

**Affiliations:** 1 Comprehensive Dentistry for Adults and Gerodontology, Faculty of Dentistry, University of Seville, 41009 Seville, Spain; evelasco@us.es (E.V.-O.); jpatomourelo@hotmail.com (J.P.-M.); dr.borjalopez@gmail.com (B.L.-L.); lomonsalve@hotmail.es (L.M.-G.); je5us@hotmail.com (J.M.-M.); enrique_aracena@hotmail.com (E.N.-M.); nunogarrido@orallagos.pt (N.M.G.); jolurr001@hotmail.com (J.L.R.-R.); ivanortizgarcia1000@hotmail.com (I.O.-G.); 2 Faculty of Dentistry, Service of the Medical-Surgical Area of Dentistry Hospital, 41009 Seville, Spain; 3 L’Hospitalet de Llobregat, University of Barcelona, 08970 Barcelona, Spain

**Keywords:** dental implants, sandblasted and acid-etched, surface, long-term follow-up, osseointegration

## Abstract

Sandblasting and acid etching are common procedures used to treat implant surfaces, enhancing osseointegration and improving clinical success rates. This clinical study aimed to evaluate the long-term outcomes of sandblasted and acid-etched implants. A total of 303 implants were placed in 114 partially and totally edentulous patients using a two-stage surgical technique and an early loading protocol (6–8 weeks). Clinical findings for implants and prosthetics were evaluated over a 12-year follow-up period. A total of 12 implants (3.9%) failed, with 3 failures occurring during the healing period before loading and 9 due to peri-implantitis. The cumulative survival rate for all implants was 96.1%. A total of 156 prostheses were placed on 300 implants, 87 single crowns, 45 partial fixed bridges, 9 full-arch fixed restorations, and 15 overdentures. The mean marginal bone loss was 1.18 mm. (SD. 0.64 mm.). Thirty-nine implants (13%) in twenty-four patients exhibited peri-implantitis. Technical complications, including prosthetic screw loosening or fracture, ceramic chipping, and acrylic fractures, were observed in 24 subjects (21.1%). Sandblasted and acid-etched surface implants placed in the maxilla and mandible reported favorable outcomes and stable tissue conditions with an early loading protocol.

## 1. Introduction

Dental implant treatment has become a therapeutic modality with a high rate of long-term success in the rehabilitation of patients with partial and total tooth loss. The development of implant dentistry has had its scientific basis in the discovery of the biological phenomena of osseointegration, which has led to the knowledge of the favorable response of hard and soft tissues to the placement of implants and their prosthetic functional load [1,2,3]. In fact, the use of implants to replace lost teeth is a very common dental treatment and is practiced by dentists around the world, both in general and specialized practice. There is a large amount of evidence on the general success of implant treatment [4,5,6,7].

Over the past 60 years, the basic principles of the osseointegration of dental implants have been established as a direct functional and structural connection between the living bone and the implant surface subjected to functional loads [8,9,10]. Studies indicate the high predictability of the osseointegration of implants, demonstrating all the typical signs of osseointegration at the bone–titanium interface through histological studies [8,9,10]. Histologically, osseointegration is characterized by the presence of newly formed bone directly interfacing with the implant surface and represents the most effective method for the long-term stabilization of dental implants. If the implant surface has a favorable microscopic structure (e.g., roughness), its bond with the host bone is increased and can activate new bone formation, penetrating the characteristic lacunae of lamellar bone on the rough surface [11,12].

Experimental and clinical research in implant dentistry has led to the development of different implant surfaces that improve the osseointegration and favor the clinical results of implants in the treatment of patients with partial and total tooth loss. From the introduction of the machined surface by Branemark et al. [8] to the more recent sandblasted and/or acid-etched surfaces, there has been an intense and prolonged research experience [10,13]. The surface of implants, or more precisely, implant surface topography, including their roughness and the orientation of surface irregularities, has been a constant concern in implant dentistry research for over 30 years. It should not be forgotten that the treatment of the surface of dental implants by increasing their roughness or developing microcavities, fissures, or cracks using various technologies can promote the union between the host bone and the implant surface [14,15].

In this sense, the macromolecules of the implant surface and the bone increase the integration of the implant with a better osteoblastic tissue response, resulting in greater resistance to compression, tension, and stress. This biological and physical response has been consistent with the results of research studies showing that this type of rough surface improves osseointegration [16,17]. Experimental studies show that a surface treated with sandblasting–acid etching increases the roughness of the implant and can improve the adhesion of osteoblastic lineage cells and have an effect on the configuration and conformation of cellular pseudopodia, which would increase cell proliferation on the implant surface [18,19,20]. This surface topography of implants appears to modulate cell growth and differentiation into osteoblasts, which affects the bone healing process, showing that surface topography can influence the phenotypic expression of osteoblasts [18,19,20].

Animal studies have demonstrated the utility of histological and histomorphometric analyses in evaluating the osseointegration of implants treated with sandblasting and acid etching [21,22,23]. Research using rabbit models highlighted the osteoconductive properties of sandblasted and acid-etched implant surfaces, showing significant bone-to-implant contact within the first 15 days post-implantation and an even greater integration at 60 days. These findings suggest that such surface treatments could accelerate implant stabilization, reduce healing time, and minimize micromotion in low-density bone areas [22]. Further supporting evidence comes from recent investigations on implants with comparable surface characteristics, which reported rapid bone integration and high levels of bone-to-implant contact after 12 weeks. These studies observed full osseointegration without infection, along with histological findings of robust bone formation surrounding the implant surfaces following the healing phase [23].

Numerous studies have demonstrated the clinical success of sandblasted–acid-etched implants in treating patients with partial or complete bone loss [24,25,26]. A study evaluated five-year radiographic follow-up results 54 patients treated with SLA (sandblasted, large-grit, acid-etched) implants [24]. Radiographs were obtained prior to the initial surgery, immediately following the first and second surgeries, at the time of final prosthesis placement, six months afterwards, and annually thereafter. The prosthetic appliances were 156 bridges and 20 single crowns. Only 14 implants reported a marginal bone loss of less than 2 mm, and 162 implants did not report marginal bone loss. Six implants failed—four in the mandible and two in the maxillaI, all in single-implant cases. The survival rate was 98.1% for implants in the maxilla and 94.3% in the mandible, resulting in an overall survival rate of 96.6% [24]. A retrospective study evaluated the decade-long outcomes of 511 titanium implants featuring a sandblasted and acid-etched (SLA) surface in 303 partially edentulous patients [26]. Over the follow-up period, the implant survival rate was 98.8%, with a success rate of 97.0%. No implant fractures were observed, although six implants (1.2%) failed. Signs of suppuration were detected in two implants (0.4%), while seven implants (1.4%) had a history of peri-implantitis [26].

This clinical study aimed to assess the long-term outcomes of sandblasted and acid-etched implants placed using a submerged surgical technique and an early loading protocol. It analyzed the implant survival rate after 12 years of loading, marginal bone loss, and the occurrence of biological and technical complications.

## 2. Materials and Methods

### 2.1. Sample Description

This research focused on individuals with partial or complete edentulism who required dental implant treatment. Prosthetic and surgical procedures took place at the Faculty of Dentistry, University of Seville, Spain, between June 2012 and April 2013. The study adhered to the ethical guidelines outlined in the Declaration of Helsinki [27] for clinical research focused on human participants. Every participant provided informed written consent for both implant placement and inclusion in this study. Ethical approval was obtained from the University of Seville’s ethics committee (No.: 002022-3, 6 June 2022).

#### 2.1.1. Demographic Distribution

A total of 114 patients participated in this study, comprising 74 females and 40 males, with ages between 26 and 71 years, with an average age of 56.4 years (SD: 12.3).

#### 2.1.2. Inclusion and Exclusion Criteria

Participants were eligible for inclusion if they were over 18 years old, had good systemic health classified as ASA I or II or managed systemic conditions effectively, and were not in need of bone regeneration procedures.

Exclusion criteria included uncontrolled chronic systemic conditions such as cardiovascular disease or diabetes, coagulation disorders, substance abuse (alcohol or drugs), smoking more than ten cigarettes daily. Participants were free of any medications or health conditions that contraindicated treatment with implants.

### 2.2. Diagnosis and Treatment Plan

**Figure 1:**
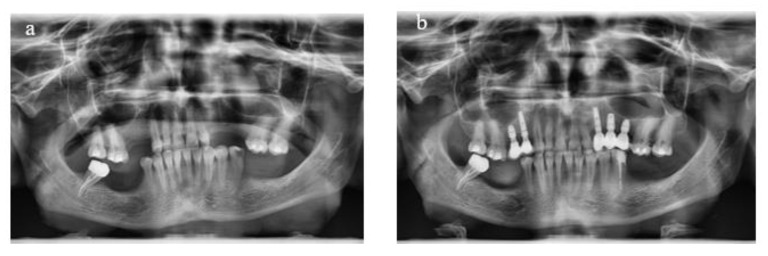
Panoramic radiographs taken before the treatment to assist in diagnosis and treatment planning (**a**) and following implant placement and prosthesis delivery (**b**).

Treatment planning involved the use of diagnostic casts to assess intermaxillary relations, along with clinical photographs and panoramic radiographs (Figure 1). Cone beam computed tomography was utilized for most patients when necessary.

### 2.3. Clinical Protocol

Every patient was administered prophylactic antibiotic therapy one hour prior to surgery, consisting of 500 mg of amoxicillin combined with 125 mg of clavulanic acid. Postoperatively, patients continued this antibiotic regimen alongside 600 mg of ibuprofen. Additionally, twice-daily use of chlorhexidine mouthwash was advised for one month. Local anesthesia with articaine and adrenaline was used for all procedures.

**Figure 2:**
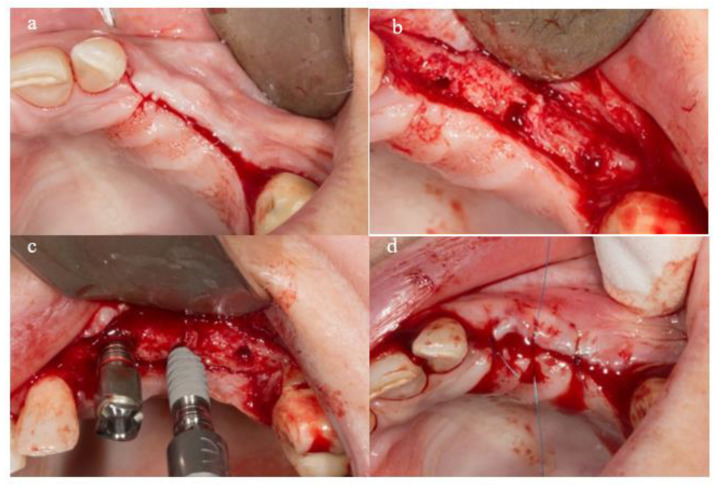
Surgical protocol. (**a**) Mucosal flap approach, (**b**) drilling of alveolar ridge (**c**) insertion of implants, (**d**) suture of mucosal flap.

A mucosal flap approach was performed, and implants were placed at predetermined sites following a prosthodontically guided treatment plan. The drilling protocol adhered to the manufacturer’s recommendations (Galimplant^®^, Sarria, Spain), where all implants were placed in healed bone using a delayed, two-stage surgical approach, with a minimum insertion torque of 35 Ncm, and without the application of bone or soft tissue grafts (Figure 2).

**Figure 3:**
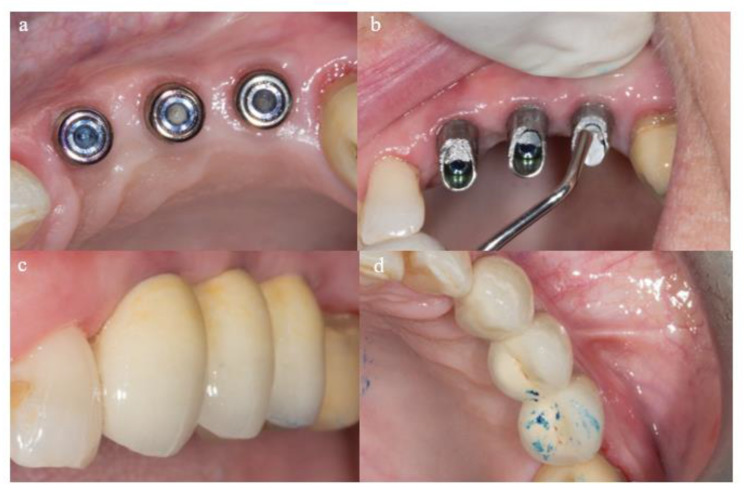
Prosthetic protocol. (**a**) Intermedial abutments, (**b**) placement of definitive abutments, (**c**) vestibular prosthesis delivery, (**d**) occlusal prosthesis delivery.

Early functional loading was performed 6 weeks after implant surgery in the mandible and at 8 weeks in the maxilla. Functional loading with definitive prostheses was completed with prosthetic abutments when the insertion torque achieved at least 35 Ncm (Figure 3).

### 2.4. Implant Characteristics

For all patients, IPX^®^ screw implants (Galimplant^®^, Sarria, Spain) were utilized. The implant surface was treated using a sandblasting and acid etching process to increase surface roughness, inducing a sub-micro topography. The implant was made of commercially pure (CP) grade IV titanium, featuring an internal hexagon connection, platform switching, V-shaped threads along the body, a conical body design, and a dome-shaped apex.

### 2.5. Follow-Up

**Figure 4:**
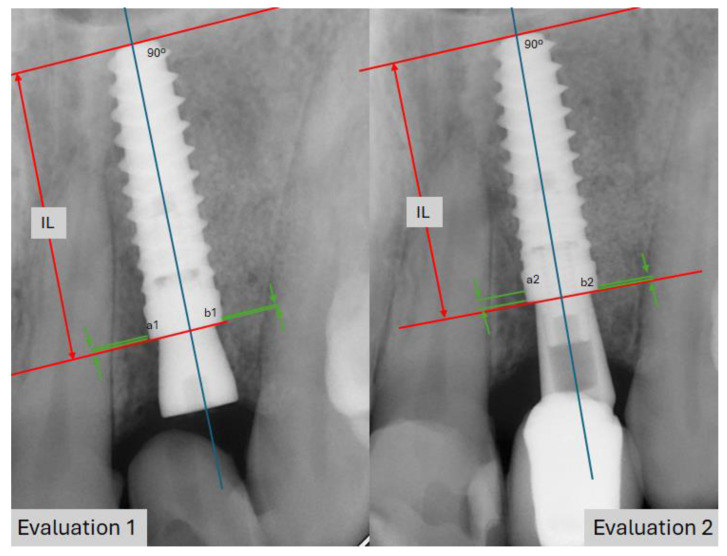
Diagram illustrating the method used to calculate mesial and distal marginal bone loss. IL: implant length. The red arrow indicates the known implant length, while the green arrows reference the crestal bone and marginal bone loss, enabling comparison at different times in this study. a1: distal bone level in evaluation 1; a2: mesial bone level in evaluation 2; b1: distal bone level in evaluation 1; b2: mesial bone level in evaluation 2.

Follow-up appointments took place at 3 and 6 months after prosthesis placement and annually thereafter, with a follow-up duration of 140.6 months on average (ranging from 120 to 148 months). Implant success was determined by its stability, along with the absence of radiolucent areas around the implant, signs of mucosal suppuration, or any associated discomfort. Marginal bone loss was measured through intraoral digital radiographs captured at a right angle to the implant’s long axis. To assess the marginal bone loss, both mesial and distal, we used the diagram shown in Figure 4. This analysis was conducted using orthopantomography and/or periapical radiographs. The length of the implant served as a reference, and measurements were taken under standardized lighting conditions using 20× magnification loupes (RS PRO Magnifier, 20× magnification, lens Ø30 mm, RS Code: 732-858, RS-PRO, Barcelona, Spain).

### 2.6. Statistical Analysis

The data were evaluated using SPSS software, version 18.0 (SPSS Inc., Chicago, IL, USA). Descriptive statistics were employed to summarize the results as mean ± standard deviation. For qualitative variables, statistical methods included the Chi-squared test and Analysis Of Variance (ANOVA). The Mann–Whitney U-test was applied to assess dichotomous variables in nonparametric tests of numerical data with non-normal distributions. A significance level of *p* < 0.05 was established.

As an example, marginal bone loss at point a2, relative to point a1, is calculated by analyzing the distortion of the implant in Evaluation 2 compared to Evaluation 1: IL2/real IL = x; IL1/real IL = y. By dividing x-y by the real IL, we determine the distortion (positive or negative) between the two radiographs. This percentage is then multiplied by a2-a1 to obtain an adjusted measurement of bone loss.

## 3. Results

A total of 303 implants were placed in 114 patients, comprising 74 females and 40 males, with partial or total edentulism. Statistical analysis revealed no significant differences related to age or sex (Chi-squared test, *p* = 0.848). Among the patients, 93 (81.6%) presented partial edentulism, 3 patients (2.6%) were totally bimaxillary edentulous, 3 patients (2.6%) totally maxillary edentulous, and 15 (13.2%) were totally mandibular edentulous. Eighteen patients (15.8%) had a previous history of periodontitis, twenty-four patients (21.1%) were smokers, and twenty-seven patients (23.7%) had a systemic chronic disease. Of the patients with a periodontal history, nine patients (50%) were smokers, and nine patients were non-smokers (50%) (chi-square test; *p* = 0.046). (Table 1). Significant statistical differences in edentulism were observed with respect to age, sex, and the follow-up period (Chi-squared test, *p* = 0.016; *p* = 0.000; and *p* = 0.000, respectively). On the contrary, previous periodontitis did not find statistical significance with age (*p* = 0.928), sex (*p* = 0.141), systemic disease (*p* = 0824), or follow-up period (*p* = 0.252), whereas a correlation was found in relation to smoking (*p* = 0.046).

Among the 303 implants placed, 129 (42.6%) had a diameter of 4.5 mm, 156 (51.5%) measured 4 mm in diameter, and 18 (5.9%) were 3.5 mm in diameter. In terms of length, 102 implants (33.7%) were 12 mm, 192 (63.4%) implants were 10 mm, and 9 (31.7%) were 8 mm. Of these, 167 implants (55.1%) were positioned in the mandible, and 136 (44.9%) were placed in the maxilla. A total of 192 implants (63.4%) were positioned in the posterior area and 111 (36.6%) in the anterior area. Twelve implants (3.9%) were lost, three during the healing phase prior to loading as a result of insufficient osseointegration and nine implants due to peri-implantitis (Table 2). The overall cumulative survival rate of the implants was 96.1%. The diameter of the implants placed did not show significance with age (*p* = 0.141) or sex (*p* = 0.111). Length was significant with respect to sex (*p* = 0.009) but not with respect to age (*p* = 0.593).

The distribution of prostheses is detailed in Table 3. A total of 114 patients received 156 prostheses, supported by 300 implants following the healing period (6–8 weeks). Specifically, 48 patients (43.1%) were treated with 48 single crowns supported by 87 implants (29%). Fixed bridges, involving 2 to 3 implants per bridge, were provided to 45 patients (39.5%), using a total of 117 implants (39%). Additionally, nine full-arch fixed restorations were delivered to six patients, with three being fully bimaxillary edentulous and three fully maxillary edentulous, supported by a total of 66 implants. Finally, 15 overdentures were provided to 15 fully mandibular edentulous patients, supported by 30 implants (Table 3).

The average marginal bone loss observed was 1.18 mm with a standard deviation (S.D.) of 0.64 mm. Values ranged from 0.6 mm to 3.5 mm over the period from implant placement to the 12-year follow-up. Patients with a history of periodontitis exhibited a higher marginal bone loss of 1.55 ± 0.48 mm compared to 1.04 ± 0.72 mm in patients without periodontitis, a difference that was statistically significant (Mann–Whitney U; *p* = 0.003). When considering smoking habits, marginal bone loss averaged 1.89 ± 1.40 mm in smokers versus 1.06 ± 0.53 mm in non-smokers, with statistically significant differences observed (Mann–Whitney U; *p* = 0.005). 

Over the follow-up period, 39 implants (13%) in 24 patients developed peri-implantitis, with 9 of these implants ultimately failing and classified as delayed failures. Peri-implantitis was significantly more common among smoking patients (87.5%) compared to non-smokers (20%), with the difference being statistically significant (Chi-squared test, *p* = 0.006).

Technical complications were observed in 24 patients (21.1%) and in 156 prostheses, including issues such as prosthetic screw loss or fracture, ceramic chipping, and acrylic fractures. Fifteen single crowns, three fixed partial bridges, and three overdentures were restored (Table 4).

## 4. Discussion

The findings demonstrated a 96.1% survival rate for early-loaded implants. A total of 114 patients were treated with sandblasted and acid-etched implants at the School of Dentistry, University of Seville, and monitored over a 12-year monitoring timeframe.

Dental implant surfaces play a key role in osseointegration. Surface roughness plays a crucial role in promoting osteogenic cell attachment and bone deposition, facilitating the formation of a sufficient bone-to-implant interface. Experimental studies have demonstrated that moderate surface roughness created optimal conditions for osseointegration. The increased surface area of moderately rough implants enhances cell attachment, supports contact osteogenesis, and encourages bone ingrowth, ultimately improving implant stability and allowing for the application of functional loading [28,29].

Sandblasting and acid-etching are among the most widely used surface treatments for enhancing osseointegration [30,31,32]. SLA surfaces have been shown to yield excellent clinical, biomechanical, and histological outcomes. This rough titanium implant surface is achieved through a combination of sandblasting and acid-etching processes. Sandblasting involves the physical application of particles such as alumina, silica, hydroxyapatite, or TiO₂, typically ranging in size from 25 to 75 µm. The type, size, temperature, and pressure of the particles significantly influence the final surface properties. Acid-etching, a chemical method for the treatment of dental surface, is commonly carried out using combinations of acids such as hydrofluoric, nitric, and sulfuric acids. The resulting surface characteristics depend on factors like the type, concentration, and duration of acid exposure [30,31,32].

In addition to the importance of the sandblasted and etched surface, an adequate macroscopic design of the implant is also important for achieving osseointegration. In this sense, the macrogeometry of the implant with its spirals and grooves is essential to achieve good primary stability. In the present study, the screw implants feature a macroscopic design suited for a two-stage surgical protocol (submerged technique) and incorporate a hexagonal internal connection. These implants have been used in several clinical studies, both in advanced cases of periodontal patients and maxillary sinus lift interventions demonstrating their clinical efficacy [33,34]. A retrospective follow-up study investigated treatment outcomes in 27 periodontal patients with immediate 305 postextraction implants for immediate rehabilitation. All patients were rehabilitated with full-arch implant-supported fixed prostheses. Clinical findings related to implants and prosthetics were assessed over an average duration of 41.3 ± 19.6 months. The success rate was 100% [33]. A long-term study was to evaluate the vertical bone gain achieved after the lateral sinus lift procedure with beta-tricalcium phosphate and simultaneous implant placement. A total of 121 sinus elevations, 81 unilateral and 20 bilateral sites, with 234 implants were performed in 101 patients. The implants were evaluated during a period of 10 years following placement. The mean vertical bone gain obtained was 6.95 ± 2.19 mm per implant after maxillary sinus augmentation. The overall implant cumulative survival rate was 97.2% [34].

In this study, the bone healing process was successful across all prosthesis types under an early loading protocol. After a healing period of 6–8 weeks, prosthodontic procedures were initiated. Abutments were placed directly onto the internal hexagonal connection for all patients, followed by the delivery of definitive prostheses. Of the prostheses provided, 90.4% were fixed restorations, including single crowns, fixed bridges, or full-arch rehabilitations, while 9.6% were removable overdentures. The clinical findings from this long-term follow-up study indicate that sandblasted and acid-etched implants placed in both maxillary and mandibular regions achieve favorable outcomes and maintain stable tissue conditions when an early loading protocol is implemented.

The development of early functional loading protocols in implant dentistry has been based on advances in implant surfaces that have increased the level of osseointegration and on new macroscopic designs that have improved their primary stability after surgery [35,36]. Several clinical studies have demonstrated the high success rate of treatment with sandblasted and etched surface implants through early loading in partially and totally edentulous patients [26,37]. A retrospective study reports that early loading after 6–8 weeks of healing in partially edentulous patients treated with sandblasted and acid-etched implants shows a survival rate of 98,8% of implants. Most implants were inserted in locations with sufficient bone volume (70%), 17.6% in narrow ridges requiring guided bone regeneration, and 12.4% in maxillary sinus lifts [26]. Similar results are also obtained for other surfaces in the work of Körmöczi K et al. [38]. A very well-structured and recent discussion (2024) on these aspects concludes that there does seem to be a relationship between the roughness of the implant, which does not affect primary stability but is directly involved in secondary stability [39].

Scientific evidence highlights the importance of bone density and cortical bone thickness in implant loading. Specifically, dental implants with optimized surface, such as sandblasted and acid-etched surface, for a rapid healing time is recommended by their mechanical, chemical and biological characteristics and for permitting a decrease in the osseointegration period. This clinical approach confirms that SLA implants can be early loaded at 6–8 weeks after placement [37]. In this sense, the findings of the current study validate the favorable clinical findings of the implants used, with a sandblasted and etched surface, as well as an internal connection with good biomechanical behavior.

On the other hand, the early and delayed loading have shown similar results in managing the success of dental implants. Although our group obtained a somewhat lower rate (93.5%) with conventional loading and other types of implants [40], a recent systematic review establishes that there are no significant differences based on the different loading times [41]. Several long-term studies have documented clinical outcomes demonstrating the success of sandblasted and acid-etched implants [38,39]. A study reported the long-term outcome of early-loaded sandblasted and acid-etched implants with segmented bridgeworks on fully edentulous maxillae. In this study, 91 implants were placed in 11 patients with edentulous maxillae. Following a 6-week healing period, abutments were secured, and fixed full-arch prostheses were cemented. Two implants failed, resulting in an implant success rate of 97.6%. Only one patient exhibited a pocket probing depth greater than 3 mm, and peri-implantitis was identified around a single implant after 4 years of functional loading. These results demonstrate a high success rate for implants and favorable peri-implant soft tissue health. [42].

A retrospective study investigated the long-term survival of dental implants in a private practice setting, reflecting routine clinical conditions. A total of 4591 sandblasted and acid-etched implants were placed in 2060 patients. Follow-up assessments were conducted at intervals of 2–3 months, 1 year, 3 years, 5 years, and 7 years, with some cases extending to 10 years. The cumulative survival rates of the implants were 99.3% at 3 years, 99.0% at 5 years, and 98.4% at 7 years. Implants placed at single sites demonstrated excellent outcomes, achieving a 100% survival rate up to 7 years. In contrast, patients with multiple implant sites showed higher failure rates. Short 6 mm implants exhibited a 100% survival rate in mandibular posterior regions but achieved only an 87% survival rate in maxillary posterior areas. Patient-related factors such as smoking, autoimmune diseases, and penicillin allergies were associated with increased rates of implant failure [43].

Marginal bone loss is a critical clinical indicator for assessing the long-term success of dental implants. Scientific evidence suggests that certain biological, material, and technical factors can influence marginal bone loss. Patient factors (smoking, diabetes), implant factors (surgery, position, macrogeometry, surface) and prosthetic factors (abutments, type of prosthetic rehabilitation) can be considered risk factors for marginal bone loss [44,45]. Several clinical studies have assessed marginal bone loss in patients treated with implants with sandblasted and etched surfaces, showing different results depending on the type of implant design, type of restorations, and duration of clinical follow-up period [24,42,43,46,47,48]. Marginal bone loss increases as the clinical follow-up period increases. A 10-year follow-up study reported a mean marginal bone loss of 0.41 ± 0.55 mm, 0.53 ± 0.43 mm, 0.68 ± 0.76 mm, and 1.01 ± 0.85 mm at the 1, 3, 5, and 10-year follow-up, respectively. Bone loss of between 3 and 4 mm was found in two patients’ three implants. None of the patients had marginal bone loss of more than 4 mm [42]. However, another clinical study reported minimal changes in marginal bone loss in implant-supported single crowns with cantilever extension in posterior areas of the maxilla and mandible after a loading time of at least 10 years. The overall mean marginal bone level changed from 0.99 mm ± 0.95 at baseline to 0.95 mm ± 0.99 at follow-up [46].

This study identified a marginal bone loss averaging 1.18 ± 0.64 mm, with a range of 0.6 mm to 3.5 mm, throughout the 12-year observation period. This level of bone loss may be attributed to the presence of risk factors, including smoking (21.1% of patients) and a history of periodontal disease (15.8% of patients). In fact, a history of periodontitis and smoking habits can be considered risk factors for marginal bone loss in patients treated with sandblasted and acid-etched implants [43,44]. A clinical study analyzed factors predicting marginal bone loss in 312 patients treated with 942 SLA implants with an average follow-up of 8.02  ±  2.5 years. Overall, the data showed a substantial stability of marginal bone loss over time, with the great majority of implants (91.1%) showing no or minimal bone loss (<1 mm) and a very low prevalence of implants presenting a bone loss ≥ 2 mm. However, a higher risk for bone loss ≥ 2 mm was observed in patients with a history of periodontitis [47]. A radiological follow-up evaluation of up to 6.5 years of 55 patients treated with 175 sandblasted and acid-etched implants reported an average marginal bone loss of 0.17 ± 0.11 mm and 0.26 ± 0.21 mm after 20 months on average and 0.53 ± 0.27 mm and 0.77 ± 0.37 mm after 81 months on average in non-smoker and smoker groups, respectively [48].

Peri-implantitis emerged as the most significant biological complication observed in this study. This condition involves inflammation of the peri-implant tissues, accompanied by progressive loss of supporting bone. Smoking and diabetes were identified as key systemic risk factors associated with peri-implant diseases. Additionally, a strong correlation was observed between peri-implantitis and a history of periodontitis [49]. In the present study, 39 implants (13%) in 24 patients (21.1%) were affected by peri-implantitis during the follow-up period, with 9 of these implants ultimately failing. A possible explanation for this high prevalence of peri-implantitis (more in reference to patients, at 21%—24 of 114 patients—than to implants, with only 13%—39 of 303 implants) may be that 183 of the implants placed (60.4%) were for multiple prostheses, the correct maintenance of which may represent difficulties for the patient.

Multiple long-term studies have confirmed the incidence of peri-implant diseases in patients treated with SLA implants [25,26,42,50]. One 10-year prospective study examined 356 sandblasted, large-grit, and acid-etched titanium dental implants in 169 fully or partially edentulous patients. Peri-implantitis was observed in 7.0% of the implants, with an incidence rate of 14.8% at the patient level over the 10-year period. During the follow-up, 12 implants exhibited adverse peri-implant symptoms requiring treatment with antibiotics, scaling, and surgical intervention. Following these procedures, all 12 cases achieved healthy and stable peri-implant conditions [25].

A prospective clinical and microbiological study with average follow-up of 10.8 years reported that peri-implant mucositis was present in around 60.2% of the implants in 73.1% of patients. The incidence of peri-implantitis affected 12.0% of implants and was observed in 15.4% of patients [46]. Subgingival microbial samples were collected from the deepest pockets around implants for bacterial analysis. Over the follow-up period, the composition of the peri-implant bacterial flora evolved in both healthy and diseased groups. A significant increase (≥20%) in bacterial frequency was noted between baseline and evaluations conducted after ≥8 years. An elevated risk of peri-implant diseases was linked to a higher concentration of specific pathogenic bacterial species [50].

In the current study, peri-implantitis was more prevalent in smoking patients (87.5%) that in non-smoker patients (20%). Implant surface can modify the disease-associated microbiome, suggesting that surface topography must be considered in the etiology of peri-implant diseases. In fact, sandblasted and acid-etched implants in patients with peri-implantitis were associated with important changes in microbiota [51]. In smokers, the incidence of peri-implantitis is increased. This condition is the result of several microbiological and immunological modifications observed around dental implants in smokers. Smoking appears to promote the adhesion of pathogen bacteria to the oral biofilm [52].

Prosthodontic complications are frequent in studies with patients treated with restorations supported by sandblasted and acid-etched implants [42,46]. The results of this study indicated technical complications in 24 patients (21.1%) out of 156 prostheses, including issues such as prosthetic screw loss or fracture, ceramic chipping, and acrylic fractures. Eighteen single crowns, three fixed partial bridges, and three overdentures had to be restored.

A clinical and radiographic study evaluating implant-supported single-unit crowns with cantilever extension in posterior areas reported technical complications after a minimum follow-up of 10 years [46]. The most common issue was loss of retention, observed in two patients (14.3%). No abutment or frameworks fractures were recorded, while the frequency of ceramic chipping and abutment screw loosening was 9.5% each [46].

The occurrence of technical complications was linked to the long-term effects of occlusal load and mechanical fatigue [42]. The most frequent technical issue reported was ceramic veneer chipping, affecting 41.7% of the prostheses. The prostheses were segmented into two pieces or four pieces in the study. Three patients required recementation at least once due to loss of retention, and one patient experienced loosening of two during the 10-year follow-up. No additional technical complications were observed throughout the study period. The success rate of prostheses was 55.3% (21 pieces out of 38 prostheses) and 27.3% at the patient level [42].

Before commenting on the limitations of our work, we would like to highlight the increasing importance that artificial intelligence should have in the management of large amounts of data, and even in using the tools provided by mathematical analysis based on the bootstrapped method [53,54]. In this way, we will have more tools to analyze the results obtained in clinical studies. For example, a previous analysis based on these tools would have allowed us to determine which patients are more prone to peri-implantitis [55], and even—with some limitations, as proposed by Bonfanti-Gris et al.—a better diagnosis of it [56].

This study has certain limitations, primarily its retrospective design. This implies that the data from the different visits were not always collected by a single researcher and that there was no control group that would allow us to compare the results. However, this also offers several strengths, including a long-term clinical follow-up, adherence to a strict surgical protocol, the inclusion of both fixed and removable prostheses, detailed documentation of complications, and comprehensive evaluation of systemic factors (e.g., smoking) and local factors (e.g., marginal bone loss) associated with implant treatment.

## 5. Conclusions

This long-term clinical study concludes that sandblasted and acid-etched implants provide a reliable option for supporting various types of prosthetic restorations, achieving high success rates for both implants and prostheses. When applied with strict selection criteria and thorough clinical planning, these implants demonstrate predictable outcomes in patients who do not require bone regeneration. In our study, a cumulative survival rate of 96.1% and a peri-implantitis rate of 13% of patients were obtained.

The observed incidence of marginal bone loss was moderate, likely influenced by factors such as a history of periodontitis and smoking, and was 1.18 mm for the period of from implant insertion to 12-year follow-up, with a range from 0.6 to 3.5 mm. The ranges of marginal bone loss were from 1.55 ± 0.48 in patients with periodontitis up to 1.04 ± 0.72 in patients without periodontitis and from 1.89 ± 1.40 in patients who smoked up to 1.06 ± 0.53 for non-smokers.

While biological and technical complications were relatively common (21.1% of patients out of a total of 156 prostheses), they did not have a significant impact on the overall success of the treatment.

## Figures and Tables

**Table 1:** Sample distribution by type of edentulism, periodontitis history, smoking habits, and systemic diseases.

Patient Description *n* = 114 (100%)		
Type of Edentulism	Total	Partial
	*n* = 21 (18.4%)	*n* = 93 (81.6%)
Periodontitis History	Yes	No
	*n* = 18 (15.8%)	*n* = 96 (84.2%)
Smoking Habits	Smoker	Non-smoker
	*n* = 24 (21.1%)	*n* = 90 (78.9%)
Systemic Diseases	Yes	No
	*n* = 27 (23.7%)	*n* = 87 (76.3%)

**Table 2:** Implant Characteristics: diameter, length, location, placement area, and preloading failure/success rates.

Implant Description *n* = 101 (100%)				
Diameter	3.5 mm 18 (5.9%)	4 mm 156 (51.5%)		4.5 mm 129 (42.6%)
Length	8 mm 9 (2.9%)	10 mm 192 (63.4%)		12 mm 102 (34.7%)
Location	Maxilla 136 (44.9%)		Mandible 167 (55.1%)	
Area	Anterior 111 (36.6%)		Posterior 192 (63.4%)	
Percentage of Failure/Success	Preloading failure 3 (1%)		Preloading success 300 (99%)	

**Table 3:** Overview of the distribution of prosthesis types in relation to the total number of patients and the implants used to support them.

Prosthesis Type	Patients *n* = 114 (100%)	Implants *n* = 300 (100%)
Single crown	48 (43.1%)	87 (29%)
Fixed bridge	45 (39.5%)	117 (39%)
Full-arch fixed	6 (5.3%)	66 (22%)
Overdenture	15 (13.1%)	30 (10%)

**Table 4:** Complications observed in implants over the 12-year follow-up.

Complication Type	+	−
Early Implant Loss	3 implants (1.5%)	300 implants (98.5%)
Delayed Implant Loss	9 implants (5.2%)	291 implants (94.8%)
Total Implant Loss	12 implants (6.5%)	291 implants (93.5%)
Peri-Implantitis	39 implants (11.8%)	252 implants (84%)
Technical complications	30 implants (19.2%)	222 implants (74%)
Average marginal bone loss: 1.18 mm. (S.D. 0.64 mm.)		

## Data Availability

There is no additional data for this work.
